# Nucleoporins and Transcription: New Connections, New Questions

**DOI:** 10.1371/journal.pgen.1000861

**Published:** 2010-02-26

**Authors:** Kohta Ikegami, Jason D. Lieb

**Affiliations:** Department of Biology, Carolina Center for Genome Sciences and Lineberger Comprehensive Cancer Center, The University of North Carolina at Chapel Hill, Chapel Hill, North Carolina, United States of America; Medical Research Council Human Genetics Unit, United Kingdom

It seems to make perfect sense that RNA, which must be exported from the nucleus to be translated, would be produced near or in association with nuclear pores. Indeed, recent reports proposed that *Saccharomyces cerevisiae* genes located close to the nuclear pore complex (NPC) tend to be highly transcribed [1],[2] and that, upon activation, some genes relocate from the nuclear interior to the nuclear periphery [3]. However, there is a critical difference in nuclear envelope organization between yeast and multicellular organisms. Yeast lacks lamins, a set of the structural proteins that coat the inner surface of the nuclear envelope, whereas multicellular organisms contain both lamins and NPCs. What is the relationship between the NPC and transcription in multicellular organisms? In this issue of *PLoS Genetics*, Vaquerizas and colleagues approach this issue [4], and in the process introduce an exciting set of new questions.

NPCs are gateways through which macromolecules are selectively imported from, or exported to, the cytoplasm. NPCs are large and highly structured protein assemblies built from more than 400 individual proteins (∼30 distinct subunits) called nucleoporins [5]. Nucleoporins and the structure of NPCs are highly conserved among eukaryotes from yeast to mammals. NPCs reside in the nuclear envelope, which is classically regarded to be associated with heterochromatin. For example, the nuclear lamina associates with transcriptionally silent regions in human and fly cells [6],[7], and artificial tethering of active genes to the nuclear lamina or to inner nuclear membrane proteins can cause transcriptional silencing [8],[9]. Indeed, electron microscopy and high-resolution light microscopy of mammalian cells clearly captures condensed heterochromatin at most of the nuclear envelope; however, heterochromatin is generally not localized at NPCs [3],[10]. So, there is an apparent paradox in the model. While the lamina interacts with transcriptionally silent loci, the NPCs, which are juxtaposed to the lamina, associate with active genes.

**Figure 1:**
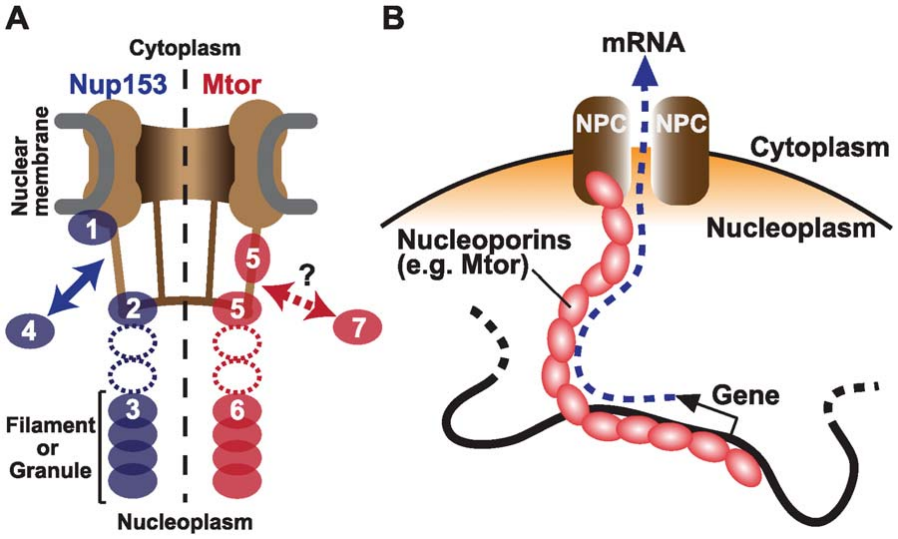
Nucleoporins Nup153 and Mtor are located at both NPCs and the nuclear interior, and associate with active transcription. (A) Schematic representation of Nup153 (left) [13]–[15] and Mtor (right) [14],[16],[17] localization at the NPC and the nucleoplasm. *Nup153* is proposed to be localized at the nuclear coaxial ring in proximity to the nuclear membrane (1); at the distal pore basket (2); as nucleoplasmic filaments (3); and shuttle between NPCs and the nucleoplasmic pool (arrow, 4). *Mtor* is proposed to constitute the pore basket (5) and nucleoplasmic filaments or granules (6). The mobile property of Mtor is unknown (7). It is still unclear whether the nucleoplasmic NUP153 and Mtor structures are extended from NPCs (dotted ovals). (B) Possible role of nucleoplasmic nucleoporins in transporting mRNA from the nuclear interior to NPCs.

The Akhtar group performed chromatin immunoprecipitation coupled with genomic tiling microarrays (ChIP-chip) and identified genomic regions associated with two nucleoporins in the fruit fly *Drosophila melanogaster*—Nup153 or Megator (Mtor), a fly homolog of mammalian Tpr (Figure 1A) [4]. While the genomic binding profiles of these two nucleoporins were similar to each other, both patterns were very different from those of typical transcription factors. Instead of being localized at discrete loci, these nucleoporins are associated with large genomic domains spanning 10–500 kb in size. These regions, named Nucleoporin Associated Regions (NARs), contain predominantly actively transcribed genes. Concordantly, within NARs the authors found high levels of RNA polymerase II binding and histone H4 lysine 16 acetylation, a modification known to relax chromatin structure in vitro [11]. The results clearly demonstrate that at least a subset of nucleoporins associate with active genes in *Drosophila*.

But do the nucleoporins associate with genes that are already active, or do they themselves promote transcription? The authors' current and previous experiments support a causal link between nucleoporins and transcriptional activation. They show that Nup153 and Mtor have a special relationship with the dosage compensation machinery. In *Drosophila*, unlike mammals and *Caenorhabditis elegans*, expression from genes on the single male X chromosome is doubled to balance expression with the two X chromosomes in female. The authors showed that NARs are over-represented on the X chromosome, but only in male cells, providing evidence for specific association between Nup153/Mtor and the active X chromosome [4]. Concordantly, these nucleoporins interact directly with a histone H4K16 acetyltransferase MOF (males absent on the first), which binds to dosage compensation complex MSL (male-specific lethal) proteins [12]. When Nup153 or Mtor were knocked down by RNAi, both MSLs and MOF dissociated from the X chromosome, resulting in reduced X-linked gene expression [12]. These findings suggest a mechanism wherein Nup153 and Mtor aid in directing the MSLs and MOF to the male X chromosome to facilitate transcription through H4K16 acetylation, further supporting an active role of nucleoporins in gene activation.

Perhaps the most surprising result is that the nucleoporins might carry out their function in transcription independent of their role in the NPC, and even independent of their localization to the nuclear envelope. By 3-D fluorescent in situ hybridization (3D-FISH), the authors determined the locations of NARs. While many NARs are found at the nuclear periphery, a subset of NARs are located at interior nuclear positions. Nup153 is known to be “mobile”, shuttling between NPCs and the nucleoplasm [13], and has been found in both the “basket” of the NPC and in filamentous structures in the nuclear interior (Figure 1A) [14],[15]. Likewise, while Mtor is localized to NPCs, it also constitutes granular or filamentous structures that extend into the nuclear interior. [16],[17]. Therefore, it is possible that the genome is associated with these internal structures to create the large NAR domains, rather than through association with the nuclear pore itself. An interesting model is that these interior nucleoporins serve as a physical route on which mRNAs are transported from deep in the nucleus to the NPC (Figure 1B) [16],[17]. This structure could be physically associated with nuclear territories or bodies to facilitate co-regulation of functionally linked genes.

Vaquerizas and colleagues clearly link a subset of nucleoporins to active gene expression and involvement with *Drosophila* dosage compensation, a chromosome-wide activation mechanism. Intriguing questions remain about how nucleoporins are targeted to specific genomic regions and the mechanism by which they affect RNA levels. The observation of both peripheral and non-peripheral NARs raises the question of whether nucleoporin-mediated regulation occurs at the NPC, in the nuclear interior, or at both locations. Finally, it remains unclear whether other nucleoporins, particularly those found exclusively as part of the NPCs, are associated with the genome or gene activity in multicellular organisms. Like any good study, this one has left us with new questions to explore.
